# Structural Properties of Molecular Sierpiński Triangle Fractals

**DOI:** 10.3390/nano10050925

**Published:** 2020-05-11

**Authors:** Eugen Mircea Anitas

**Affiliations:** 1 Joint Institute for Nuclear Research, Dubna 141980, Russia; anitas@theor.jinr.ru; 2 Horia Hulubei, National Institute of Physics and Nuclear Engineering, 077125 Bucharest-Magurele, Romania

**Keywords:** structural properties, small-angle scattering, form factor, structure factor, fractals, Sierpiński triangles

## Abstract

The structure of fractals at nano and micro scales is decisive for their physical properties. Generally, statistically self-similar (random) fractals occur in natural systems, and exactly self-similar (deterministic) fractals are artificially created. However, the existing fabrication methods of deterministic fractals are seldom defect-free. Here, are investigated the effects of deviations from an ideal deterministic structure, including small random displacements and different shapes and sizes of the basic units composing the fractal, on the structural properties of a common molecular fractal—the Sierpiński triangle (ST). To this aim, analytic expressions of small-angle scattering (SAS) intensities are derived, and it is shown that each type of deviation has its own unique imprint on the scattering curve. This allows the extraction of specific structural parameters, and thus the design and fabrication of artificial structures with pre-defined properties and functions. Moreover, the influence on the SAS intensity of various configurations induced in ST, can readily be extended to other 2D or 3D structures, allowing for exploration of structure-property relationships in various well-defined fractal geometries.

## 1. Introduction

Deterministic nano and micro scale fractals have many exciting and unusual properties with important potential uses in microwave/radio frequency antenna design [1], coordination chemistry [2] or stretchable electronics [3]. Many bulk properties of synthesized deterministic fractals depend upon their structural properties. Therefore, understanding the relative arrangement, shape and size of the basic units is very useful for specific applications. In particular, Sierpiński carpets [4] provide an efficient structure for the realization of compact active devices with a strong and broadband spectral response in the visible/mid-infrared range [5], terpyridine-based supramolecular structure such as Sierpiński triangles (ST) or hexagonal gaskets are widely applied in catalytic processes [2], or 3D octahedral-like fractals can act as effective structures for gas permeation in micro-channels [6].

Fabrication of such structures is a complex process involving an interplay between halogen, hydrogen, metal-organic coordination and covalent bonds, depending on the specific type of atoms used to build the fractal, as well as on the substrate [7]. Up to now, only ST fractals can be prepared on surfaces in a controlled way [8]. As such, ST fractals are constructed on both Ag(111) and symmetry-mismatched fourfold Ag(100) surfaces through chemical reactions between H3PH molecules and Fe atoms in vacuum [9]. ST prisms can be built by dynamic complexation reactions between tetratopic cavitand-based ligand and various multitopic counterparts in the presence of Cd${}^{\mathrm{II}}$ ions [10]. Furthermore, 2D STs are also obtained by the synthesis of a covalent organic framework based on the low-symmetry 1,3-benzenediboronic acid precursor [11]. Sierpiński pyramids are assembled by lipophilic-lipophilic association of alkyl moieties and a complementary perfect fit of ST based on terpyridine metal complexes with alkylated corners [12]. Self-similar molecules with symmetrically backfolded shapes inspired by ST are synthesized based on phenylalkynyl or on molecules with linear backbones on Au(111) in which the coordination nodes between the metal atoms and organic ligands are formed via coordinate interactions [13]. Electronic Siepriński fractals are formed from arrays of artificial atoms defined by controlled position of CO molecules on a Cu(111) surface [14]. STs based on Ag(111) surface below 80 K is achieved by formation of synergistic halogen and hydrogen bonds between aromatic bromo compounds [15].

However, generally, the resulting STs obtained from the existing experimental methods, are not idealized structures in the mathematical sense, and thus modeling their structure with a standard ST model may introduce some undesired artefacts. Such deviations from the idealized model, include small displacements of the basic units (building molecules), such as for STs on symmetry mismatched Ag(100) [9] and on Au(100)-(hex) surfaces [16], different shapes of the basic units [15,17], or inclusion of additional molecules to act as bonds between the basic units [12]. In the later approach, the distances between the building blocks is increased while their size is kept unchanged. All these modifications induce various physical effects, which can be rigorously described, once the exact structure of ST is determined.

Here, the structural changes induced by these deviations are investigated by performing a detailed analysis of the corresponding small-angle scattering (SAS) curves. To this aim, in the first part of the paper are derived analytic expressions for the form and structure factors of ideal STs. Then, the model is extended to include various sizes of STs, different ratios between the size of the basic units and distances between them, and small displacements of the basic units. The influence of the shape of basic units on the scattering curves is also discussed, and in anticipation of hierarchical ST-based fractals, the SAS curves of ST in which the basic units are themselves fractals are investigated. It is shown that while some parameters such as the fractal dimension or the overall sizes of ST, and respectively of the basic units, can be still recovered for any type of structural change, some other parameters, such as the scaling factor can be hardly obtained when the size polydispersity of STs is high enough.

The influence of the structural changes presented here can readily be extended to extract structural information from other 2D/3D molecular fractals, such as Sierpiński carpets, Vicsek fractals [18], Menger sponges [19], hydrant surfaces [20] or fat fractals [21], with arbitrarily shapes of the basic units.

## 2. Theoretical Background

### 2.1. Small-Angle Scattering Method

One considers a beam of either neutrons or X-rays incident on a sample of volume $V^{{}^{\prime }}$, which consists from a macroscopic number of objects with scattering length $b_{j}$. Thus, the scattering amplitude A(q) can be written as [22] $A\left(q\right)=\int_{V^{{}^{\prime }}}\rho_{s}\left(r\right)e^{-iq\cdot r}d^{3}r$, where q is the scattering vector. Here, $\rho_{s}\left(r\right)=\sum_{j}b_{j}\delta \left(r-r_{j}\right)$ is the scattering length density (SLD), $r_{j}$ are the positions of the scatterers, and δ is the Dirac δ-function. As such, the differential cross section of the irradiated sample is given by $d\sigma /d\Omega ={\left|A\left(q\right)\right|}^{2}$.

It is also assumed that the sample is a two-phase system composed from homogeneous units of “mass” density $\rho_{m}$ embedded into a solid matrix of “pore” density $\rho_{p}$. Then, by subtracting the “pore” density, we deal with a system in which the objects of density $\Delta \rho =\rho_{m}-\rho_{p}$ are “frozen” in a vacuum. This holds true, since a constant density shift across the sample plays a role only at small values of *q*, which however, are usually beyond the instrumental resolution. If the sample consists of many identical objects of volume *V* with concentration *n* and whose spatial positions and orientations are uncorrelated, the scattering intensity can be written as [23]:

(1) $$I\left(q\right)\equiv \frac{1}{V^{\prime }}\frac{d\sigma }{d\Omega }=n{\left|\Delta \rho \right|}^{2}V^{2}\langle {\left|F\left(q\right)\right|}^{2}\rangle ,$$

where Δρ is called the scattering contrast, and the normalized scattering amplitude (form factor) of the scattering object is written as:

(2) $$F\left(q\right)\equiv \frac{1}{V}\int_{V}e^{-iq\cdot r}dr.$$

Thus, at q=0, the forward scattering intensity becomes:

(3) $$I\left(0\right)={n|\Delta \rho |}^{2}V^{2}.$$

In Equation (1), the notation $\langle \cdots \rangle$ denotes the ensemble averaging over all orientations. Explicitly, in 2D, it can be calculated as:

(4) $$\langle f(q_{x},q_{y})\rangle =\frac{1}{2\pi }\int_{0}^{2\pi }f\left(q,\phi \right)\;d\phi ,$$

where $q_{x}=q\cos\phi$ and $q_{y}=q\sin\phi$.

The most important properties of the form factor, which will be used here, are:

1. F(q)→F(βq) when the length of the particle is scaled as L→βL,
2. $F\left(q\right)\to F\left(q\right)e^{-iq\cdot a}$ when the particle is translated r→r+a,
3. $F\left(q\right)=\left[A_{I}F_{I}\left(q\right)+A_{II}F_{II}\left(q\right)\right]/\left(A_{I}+A_{II}\right)$, when the particle consists of two non-overlapping subsets *I* and II with surface areas $A_{I}$ and $A_{II}$.

At *m*-th iteration, the fractal consists from:

(5) $$N_{m}=k^{m},$$

units of the same shape and size, where *k* is the rate of increase of the number of units at each iteration. Then, the corresponding form factor can be written as [23]:

(6) $$F\left(q\right)=\rho_{q}F_{0}\left(q\right)/N_{m},$$

where $F_{0}\left(q\right)$ is the form factor of the basic unit, and $\rho_{q}=\sum_{j}e^{-iq\cdot r_{j}}$ is the Fourier component of the density of the scattering centers, with $r_{j}$ being their positions.

For an Euclidean object of size *L* embedded in a*d*-dimensional space, the normalized SAS intensity can be written as: [24]

(7) $$\langle |F_{0}{\left(q\right)|}^{2}\rangle ≃\{\begin{array}{cc} 1, & q≲2\pi /L,\\ {\left(qL/2\pi \right)}^{-\tau }, & 2\pi /L≲q, \end{array}$$

with τ=d+1, and which consists of two main structural regions: the Guinier region (q≲2π/L) and the Porod region (q≳2π/L), from which we can extract information about the overall size of the object, and respectively about its specific surface.

By denoting with $F_{0}$ the form factor of the initiator, Equation (1) gives [23]:

(8) $$I\left(q\right)=I\left(0\right)S\left(q\right)|F_{0}{\left(q\right)|}^{2}/N_{m},$$

where S(q) is the fractal structure factor defined by:

(9) $$S\left(q\right)\equiv \langle \rho_{q}\rho_{-q}\rangle /N_{m}.$$

This is related to the pair distribution function and provides information about the relative positions of the scattering centers inside the fractal. Equation (9) can be rewritten as:

(10) $$S\left(q\right)=\frac{1}{N_{m}}\sum_{j,k=1}^{N_{m}}\langle e^{-iq(r_{j}-r_{k})}\rangle ,$$

where the structure factor at zero angle is $S\left(0\right)=N_{m}$.

Physical objects, almost always have different sizes. Here, one assumes that they have the same shape, but their sizes vary and obey a statistical distribution $D_{N}\left(l\right)$ defined in such a way that $D_{N}\left(l\right)dl$ gives the probability of finding an object whose size falls within $\left(l,l+dl\right)$. Without losing generality, one considers here a log-normal distribution of the type:

(11) $$D_{N}\left(l\right)=\frac{1}{\sigma l{\left(2\pi \right)}^{1/2}}e^{-\frac{{\left(\ln\left(l/l_{0}\right)+\sigma^{2}/2\right)}^{2}}{2\sigma^{2}}},$$

where $\sigma ={\left(\ln(1+\sigma_{r}^{2})\right)}^{2}$ is the variance and $l_{0}={\langle l\rangle }_{D}$ is the mean value of the length, $\sigma_{r}\equiv {\left({\langle l^{2}\rangle }_{D}-l_{0}^{2}\right)}^{1/2}/l_{0}$ is the *relative* variance, and ${\langle \cdots \rangle }_{D}=\int_{0}^{\infty }\cdots D_{N}\left(l\right)dl$. Therefore, the corresponding scattering intensity of a polydisperse system is obtained by averaging Equation (1) over the distribution function (11) according to [22]:

(12) $$I\left(q\right)={n|\Delta \rho |}^{2}\int_{0}^{\infty }\langle {\left|F\left(q\right)\right|}^{2}\rangle S_{T}^{2}\left(l\right)D_{N}\left(l\right)dl.$$

where $S_{T}$ is the surface area of the ST.

### 2.2. Form Factor and Scattering Intensity of a Triangle

In calculating the form factor, start from an isosceles triangle. Then, equilateral triangles, as those used in ST, are obtained in the limiting case when all the edges are equal. Thus, for an isosceles triangle of base length *l*, its height *h* is given by $h=l\sqrt{3}/2$. Let us consider a Cartesian coordinate system with the ox-axis parallel to the base, and the opposite vertex coinciding with the origin. The corresponding form factor can be written as [25]:

(13) $$F_{0}\left(q\right)=\frac{1}{S_{T}}\int_{0}^{l}dy\int_{-yl/(2h)}^{yl/(2h)}e^{-i(q_{x}x+q_{y}y)}dx,$$

where $q=\{q_{x},q_{y}\}$. Performing the calculations in Equation (13) one obtains an analytic expression for the form factor, given by [25]:

(14) $$F_{0}\left(q\right)=2e^{-\alpha }\frac{\beta e^{i\alpha }-\beta \cos\beta -i\alpha \sin\beta }{\beta \left(\beta^{2}-\alpha^{2}\right)},$$

where: $\alpha =hq_{x}$ and $\beta =hq_{x}^{2}/2$.

## 3. Results and Discussion

### 3.1. ST Model

**Figure 1:**
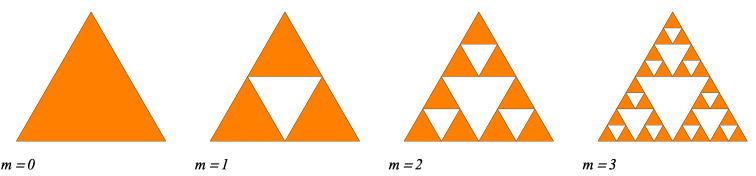
Initiator (m=0), and first three (m=1,2,3) iterations of the Sierpiński triangle fractals.

The standard ST fractal is constructed from equilateral triangles, as shown in Figure 1 for the first four iterations. It is considered that at iteration m=0, the triangle is centered in the origin. If we denote its edge length by *l*, then its area becomes $S^{T}=\sqrt{3}l^{2}/4$. Then, m=1 the triangle is divided into four smaller triangles, each of edge length l/2, in which the three triangles in the corners are kept, while the middle one is removed, as shown in Figure 1. When m=2, the same operation is repeated for all three triangles of edge length $l_{1}\equiv l/2$. The ST fractal is obtained in the limit of high number of iterations. The number of triangles composing the ST at an arbitrary iteration number *m*, is given by Equation (5) with k=3, i.e.,

(15) $$N_{m}=3^{m}.$$

Similarly, the edge length of each triangle at *m*-th iteration can be written as:

(16) $$l_{m}=l/2^{m}.$$

Therefore, by using Equations (5) and (16), the fractal dimension gives [23]:

(17) $$D=\lim_{m\to \infty }\frac{\log N_{m}}{\log\left(l/l_{m}\right)}\approx 1.58.$$

Note that since all the triangles composing the ST have the same size, the structure is a mass fractal. Therefore, the scattering exponent in the scattering curve shall coincide with the the analytical value of fractal dimension given by Equation (17) (see also [23,26,27]).

### 3.2. Extensions of ST Model

**Figure 2:**
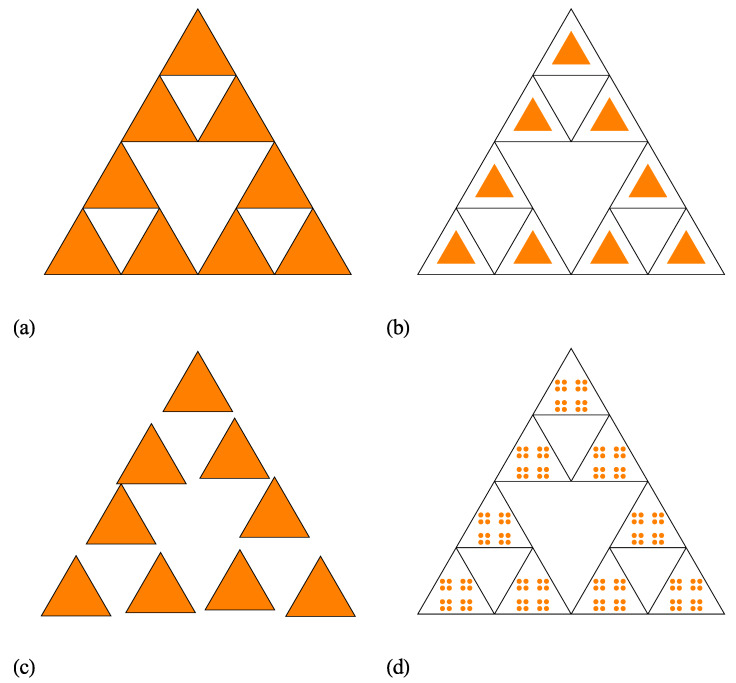
Various models of Sierpiński triangle (ST) fractals at iteration m=2. (**a**) Regular ST (model ST). (**b**) Scaled ST (model SST). (**c**) Randomised ST (model RST). (**d**) Complex ST (model CST).

As a first extension of the regular ST model, let us consider that the distances *h* between the scattering units within a cluster, are much higher than the overall size (≃l), i.e., h/l≫1. For the regular ST, shown also in Figure 2a at iteration number m=2, we have h/l≃1. A representative example with h/l≫1 at m=2 is shown in Figure 2b, and this model is called the scaled ST (SST). However, in Figure 2b the sizes of the basic units of SST are smaller than those of ST, while their positions are unchanged. In particular, for a good visual representation, here the size of basic units was chosen such that h/l≃2. Since, both the number of basic units at an arbitrarily iteration number *m* and the ratio $l/l_{m}$ for the SST model are unchanged as compared to the ST model, the fractal dimension (given by Equation (17)) remains also unchanged. In addition, while the overall size of SST remains approximately the same as for the regular ST, the size of the basic units is significantly reduced. As we shall see below, all these features are reflected in the behaviour of the scattering intensity.

A second extension model of the regular ST is shown in Figure 2c (also at m=2). Here, the sizes of the basic units are kept unchanged, while their positions are allowed a small displacement, such that they do not overlap. This is called the randomized ST (RST) model. It is clear that in the limiting case, when the positions of the basic units are allowed arbitrarily positions, but still keeping the condition of non-overlapping, the scattering intensity is reduced to the one of a collection of triangles with uncorrelated positions, with the corresponding factor given by Equation (14). The overall size of the fractal is approximately the same as for ST and SST models, while the sizes of the basic units are the same as for the ST model.

The last extension of ST models presented here, involves changing the shape of the basic units. This has important effects on various properties, such as the specific surface, and is reflected in the Porod region of the scattering curve. It is well-known that by choosing another Euclidean shape of the basic unit, instead of the triangle, the changes in the values of the specific surface may not be enough pronounced. However, by changing the shape of the triangle, also into a fractal, the specific surface (as well as other properties) can be drastically changed at various scales. Figure 2d shows an example in which the shape of the triangle is replaced by a well-known Cantor fractal [28] (here, shown at iteration n=2). This is called a complex ST (CST) model. However, generally instead both the ST and the Cantor fractal, one can consider any other fractal structures. Although, according to the author’s knowledge there is no experimental realization of such complex structures, it is expected that they shall play an important role in various applications which require a combination of different properties at different scales in a single structure.

### 3.3. Structure and Form Factors

For determination of the structure and form factors of the ST, it is followed an approach similar to the one used in Reference [26], and a product of the generative functions $G_{i}\left(q\right)$ of ST at various iterations, is introduced as:

(18) $$P_{m}\left(q\right)\equiv \prod_{i=1}^{m}G_{i}\left(q\right).$$

Here, the generative function at iteration m=1 is written as:

(19) $$G_{1}\left(q\right)=\frac{1}{3}\sum_{k=0}^{2}e^{-iq\cdot b_{k}},$$

while the generative function at an arbitrarily iteration *m* satisfies the relation $G_{m}\left(q\right)=G_{1}\left(\beta^{m-1}q\right)$. In the above relation, the translation vectors are defined as:

(20) $$b_{k}=\frac{a\sqrt{3}}{6}\left\{\cos\left(\frac{\pi }{3}\left(2k+\frac{3}{2}\right)\right),\sin\left(\frac{\pi }{3}\left(2k+\frac{3}{2}\right)\right)\right\}.$$

The scaling factor for regular ST used here is β=1/2. By using Equation (18), we can write that $\rho_{q}^{\left(m\right)}=N_{m}P_{m}\left(q\right)$, and from Equation (9), we recover the structure factor (see also Reference [26]):

(21) $$S_{m}\left(q\right)/N_{m}=\langle \prod_{i=1}^{m}{\left|G_{i}\left(q\right)\right|}^{2}\rangle ,$$

where $G_{1}\left(q\right)$ is given by Equation (19). Then, the total intensity, or the fractal form factor, is obtained by using the expression of the triangle form factor given by Equation (14) in Equation (8).

**Figure 3:**
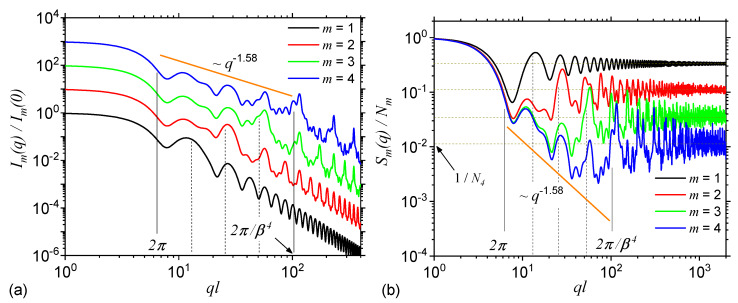
Form (**a**) and structure factors (**b**) of Sierpiński triangle (ST) at first four iterations. For the form factor, the curves corresponding to m≠1 are shifted vertically, for clarity, by a factor of $10^{m-1}$. The continuous vertical lines denote the beginning and respectively the end of the fractal region at m=4. The dashed vertical lines denote the end of the fractal region at iterations m=1,2 and 3. *l* is the overall size of the ST, β=1/2 is the scaling factor, and $N_{4}=3^{4}$ is the number of triangles composing the ST at m=4.

Figure 3a shows the SAS intensities from the first four iterations of the regular ST calculate with Equation (8) and the basic unit form factor given by Equation (14). The results show that each curve is characterized by the presence of a Guinier region at q≲2π/l. Its length is approximately the same for each iteration and it provides a measure of the ST’s overall size. At $2\pi /l≲q≲2\pi /\left(\beta^{m}l\right)$ one can observe a complex superposition of maxima and minima on a power-law decay, called a generalized power-law decay (GPLD) [23]. It is clear from the right hand side of the above approximate inequality that the upper end of this region increases with the iteration number, and it gives a measure of the overall size of the basic units composing the fractal. The higher the iteration number, the smaller their size and thus the end of this region is more shifted to the right. Since here, the scattering exponent gives the fractal dimension of ST, this is called a fractal region. Note that the fractal dimension obtained from SAS curve is in very good agreement with the analytical one obtained by using the analytic formula in Equation (17). However, this implies that the SAS curves are calculated at least for the first three iterations. Furthermore, Figure 3a shows that in the fractal region, the number of the most pronounced minima coincide with the iteration number, while the periodicity is related to the ST’s scaling factor. Finally, at $q≳2\pi /\left(\beta^{m}l\right)$, the Porod region occurs, which can be used to extract the specific surface of ST [22].

Figure 3b shows the structure factors of STs also at first four iterations, calculated by using Equation (21), with the generative function at m=1, given by Equation (19). Since in Equation (21) the form factor of the basic units is omitted, the Porod region, specific to the ST fractal form factor is replaced by an asymptotic one in which $S_{m}\propto 1/N_{m}$. Thus, the values of these asymptotics can be used to obtain the number of the basic units at a given iteration number. Note that since the difference between the fractal form and structure factors is the presence, in the former case, of the basic unit form factor, while its influence appears only in the Porod region [29], it turns out that in both cases the Guinier and fractal regions shall be very similar. This can be clearly seen in the behaviour of scattering curves in Figure 3a,b, and where both the number and the periodicity of minima in the fractal region as well as the limits and slope of the GPLD remain unchanged.

### 3.4. Influence of Various Sizes of the Basic Units Relative to the Distances Between Them, and of Polydispersity

**Figure 4:**
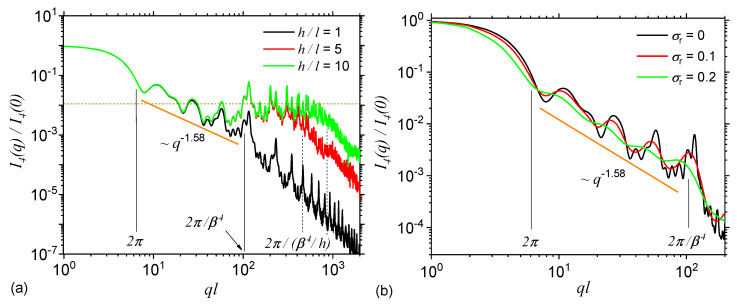
Form factor of ST at m=4. (**a**) Monodispere curves, with various values of the ratio h/l of the distances between triangles to their size. (**b**) Polydisperse curves, with various values of the relative variance $\sigma_{r}$. *l* is the overall size of the ST, and β=1/2 is the scaling factor.

By using the property 1 listed after Equation (4), with the replacements l→l/5, and respectively l→l/10 (or equivalently, considering h/l≃5, and respectively h/l≃10) in Equation (14), then the scattering intensity at iteration number m=4, given by Equation (8) gives the curves shown in Figure 4a in red, and respectively in green colours. For comparison, the scattering intensity at h/l≃1 is also shown (black colour). The results show that when the ratio h/l>1, a second plateau, where $I\left(q\right)\propto q^{0}$ occurs at $q≳2\pi /\left(\beta^{4}l\right)$, which is then followed by a Porod region at $q\equiv q_{h}≳2\pi /\left(\beta^{4}l/h\right)$. The last two relations, show that the length of this plateau is proportional to the typical distances *h*, while its asymptotic value is proportional to the number of basic units composing the ST, as shown also in Figure 3b. Therefore, when $q≲q_{h}$ the scattering intensity is dominated by the structure factor given by Equation (21), while for $q≳q_{h}$ the dominant contribution is that of the triangle form factor given by Equation (14) with its edge length properly rescaled, i.e., l→l/5 and l→l/10 in Figure 4a. Thus, experimentally, the length of this plateau can be used to estimate the relative size of the typical lengths between fractal clusters in ST, relative to the size of their edge length.

The effects of size polydispersity are taken into account by using Equation (12) at various values of the relative variance: $\sigma_{r}=0.1$ (red curve) and $\sigma_{r}=0.2$ (green curve) at iteration number m=4. For comparison, the complete monodisperse intensity is also included (black curve). The results show that the scattering curves become smoother with increasing the relative variance, and the maxima and minima present in the fractal region dampens out. Therefore, in experimental situations, if the polydispersity of the STs is high enough (i.e., $\sigma_{r}≳0.4$), the periodicity of the intensity in the fractal region can be hardly noticed, and thus extracting the fractal iteration number and the scaling factor of the ST can become a difficult task. However, the slope of the scattering curve in the fractal region remains unchanged, and therefore the fractal dimension (D≃1.58) can be still recovered. Furthermore, the limits of the fractal region are slightly shifted to the left, indicating that the used polydispersity generates, on the average, slightly higher sizes of the STs and of its composing units, as compared to the monodisperse case.

Note that from a practical point of view, such smooth curves are a rule rather than exception. This is because, as for now, it is quite difficult to prepare systems containing complete monodisperse fractals, and also due to the limited resolution of the spectrometers. However, further developments in both sample preparation of deterministic fractals as well as improvements in instrumentation are expected to overcome, at least partially, such difficulties in recovering the iteration number and the scaling factor.

### 3.5. Influence of Small Random Displacements and of the Shape of Basic Units

**Figure 5:**
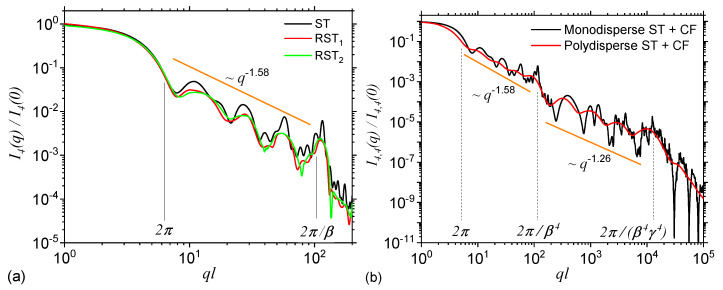
(**a**) Form factor of ST, and two randomized versions of ST (RST${}_{1}$ and RST${}_{2}$) as shown in Figure 2c at iteration m=4. In the model RST${}_{2}$ the positions of triangles is allowed a higher degree of freedom as compared to model RST${}_{1}$. (**b**) Form factor of a complex fractal consisting from disks forming a Cantor fractal at iteration n=4, which in turn is arranged as the triangles in a ST fractal, also at iteration m=4 (see Figure 2d).

The effect of small random displacements of the basic units from the positions characteristic to a regular ST is shown in Figure 5a at iteration number m=4, and for two random configurations RST${}_{1}$ (red curve) and RST${}_{2}$ (green curve). Here, in the model RST${}_{2}$ the degree of randomness allowed is higher as compared to the one in model RST${}_{1}$. For comparison, in the same figure is included also the scattering intensity without any randomness (black curve). The results show that the shape of maxima and minima are changed, and their amplitudes are decreased. This leads to a partial smoothing of the curve in the fractal region, similar to the behaviour seen in Figure 4b when size polydispersity is included. However, as opposed to the later case, in the former one, a small displacement of the basic units indicate a decreasing of the slope in the fractal region. While the smoothing can be attributed to the fact that the degree of correlations between the positions of the center-of-masses decreases, the decrease of the slope arise due to an increasing influence of the contribution from individual triangles. One expects that when the correlations between the center-of-masses are completely removed, the scattering intensity to resemble, up to a constant factor, that one of a triangle. Note that the lengths of the Guinier and fractal regions remain practically unchanged with introducing small random displacements, and this feature can be used to differentiate RST models from ST with polydispersity.

The effect of introducing a fractal shape for the basic units of ST is shown in Figure 5b, where the triangles are replaced by mass Cantor fractals at iteration number n=4. The back curve corresponds to the monodisperse case, while the red one to a polydisperse case with $\sigma_{r}=0.2$. The construction and scattering properties of Cantor fractals have been presented elsewhere [25]. The scaling factor used is γ=1/3, the number of particles at n=0 is also four, and thus the corresponding fractal dimension is ≃1.26. For this complex configuration, the fractal region corresponding to ST is immediately followed at $q≃2\pi /\left(\beta^{4}l\right)$ by the fractal region of the Cantor fractal, up to $q≃2\pi /\left(\beta^{4}\gamma^{4}l\right)$, and only then it follows a Porod region corresponding to the basic units of the Cantor fractal (here disks). From this second fractal region, one can extract also the iteration number and scaling factor by using the same procedure as for STs. Such a succession of fractal regimes is specific to hierarchical systems, with structures at multiple levels as in particles-cluster-aggregates clusters.

A similar behavior can be obtained when the Cantor fractal is replaced by a surface fractal, such as 2D Sierpński carpet, and which is also widely used in various applications, such as in the design of fractal antennas [5]. In this case, the mass fractal region of the Cantor fractal is replaced by a surface fractal region in which the intensity decays as $I\left(q\right)\propto q^{D_{\mathrm{SC}}-4}$, where $D_{\mathrm{SC}}≃1.89$ is the fractal dimension of the Sierpiński carpet. In this case one can also recover the same structural information as for mass Cantor fractals, although the nature of some features, such as the periodicity of the scattering curve in the fractal region, may be different (see also Ref. [25] for more details).

Experimentally, the behaviour of the scattering curves is influenced to a certain degree by the instrumental limitations that arise from the finite resolution of the detection systems and from the wavelength spread of the incident radiation. They lead to a smearing of the scattering curve, in a similar way polydispersity does [30]. In the case of deterministic fractals, such as the STs investigated here, one may have either a partial or a complete smoothing of maxima and minima. In the first case, when the periodicity of maxima and minima in the fractal region can be still observed, one can recover all the structural information discussed above. Note also that the q-independent background of the intensity, and which is determined by the scattering density of irradiated nuclei with nonzero spins such as 1 H or 7 Li isotopes, may hinder the fine structure of SAS curve at high q values. To avoid such issue, one can use deuteration to increase the contrast and reduce the background.

## 4. Conclusions

In this work have been presented analytic expressions for the form and structure factors of 2D STs (Figure 2a). In order to model accurately the structure of newly created deterministic STs, several extensions of the ST model have been proposed: a scaled version, in which the sizes of the composing triangles are scaled down while the distances between them are kept unchanged (model SST; Figure 2b), a randomised version, in which the triangles composing the ST are allowed small random displacements but without overlapping (model RST; Figure 2c), and a complex version in which the triangles are replaced by Cantor fractals (model CST; Figure 2d).

For all the extensions introduced, the corresponding SAS intensities have been calculated and the influence of different parameters on the scattering curve has been analyzed. It has been shown that the form factor corresponding to the standard ST model is characterized by the presence of three main regions: Guinier (at low *q*), fractal (at intermediate *q*), and Porod (at large *q*). Each of these regions provide valuable information about the structure of ST: the overall size (from the end of the Guinier region), fractal dimension, iteration number and the scaling factor (from the GPLD in the fractal region), and the specific surface (from the Porod region). It has been also shown that the corresponding structure factor of STs has a similar behaviour as the form factor, excepting the Porod region, which is replaced by an asymptotic one, with the values related to the total number of triangle composing the ST. As compared to the ST’s form factor, in the SST model, an additional plateau (i.e., a region with $I\left(q\right)\propto q^{0}$) occurs between the fractal and Porod regions. Its length depends on the factor by which the triangles composing the ST are scaled down. In the RST model, the curves become smoother and their slope decrease with increasing the degree of displacements of the triangles. In the CST model, a succession of two GPLD occur: the first one corresponds to STs, and the second one to the Cantor fractal. They are followed by the Porod region of the basic units of the Cantor fractal. The second GPLD can be used to extract the same type of structural information about Cantor fractals, in the same manner as the first GPLD is used for STs.

The obtained results can be used to analyze experimental SAS (x-rays or neutrons) data from ST fractals which include various deviations from the ideal structure of deterministic ST fractals. The range of structural parameters which can be extracted within the suggested approach allows the design and fabrication of well-defined molecular fractal structures with predefined properties and functions.
